# Recent Advances in Protein Tyrosine Sulfation and Its Emerging Roles in Human Diseases

**DOI:** 10.3390/cells15211985

**Published:** 2026-10-30

**Authors:** Ning Liu, Xiaowen Chen, Hailong Zhao, Bo He

**Affiliations:** 1 Department of Pathophysiology, School of Preclinical Medicine, Zunyi Medical University, Zunyi 563000, China; 2 Research Center of Humanities and Medicine, School of Management, Zunyi Medical University, Zunyi 563000, China; 3 West China School of Basic Medical Sciences & Forensic Medicine, Sichuan University, Chengdu 610041, China

**Keywords:** protein tyrosine sulfation, post-translational modification, biological functions, regulatory mechanisms, therapeutic targets

## Abstract

Protein tyrosine sulfation (PTS), an important post-translational modification (PTM), has garnered increasing attention in life sciences in recent years. Following classical and emerging PTMs such as phosphorylation, lactylation, acetylation, and palmitoylation, PTS has now emerged as a novel entry point in disease mechanistic studies. As an evolutionarily conserved modification ubiquitously present in organisms, PTS acts as a core molecular switch that modulates fundamental biological processes via specific conjugation of sulfate groups to tyrosine residues of target proteins. Accumulating evidence has revealed that PTS modifies numerous pivotal functional proteins, such as chemokine receptors, histones, and coagulation factors, and participates in multiple physiological and pathological events, including hormonal modulation, gene transcription, hemostasis, inflammatory responses, cancer progression, and viral infection. Consequently, deeper insight into the mechanisms of PTS and its targeted intervention holds promise for novel therapeutic strategies for related diseases. Nevertheless, the molecular regulatory network of PTS remains poorly understood, which is largely attributed to the limitations of current techniques for the efficient preparation and accurate detection of PTS-modified proteins. This review systematically summarizes the latest advances in the biological functions and regulatory mechanisms of PTS, aiming to provide a theoretical reference for subsequent fundamental research and offer potential therapeutic targets for the clinical treatment of PTS-related diseases.

## 1. Introduction

Proteins serve as the central functional executors of nearly all cellular biological activities, and their functions are tightly governed by versatile post-translational modifications (PTMs) throughout their entire life cycles [1,2,3]. PTMs represent a pivotal layer of post-genomic regulation that profoundly remodels protein conformational structure, subcellular localization, stability, and enzymatic activity. Through these multifaceted regulatory manners, PTMs dramatically enrich the compositional complexity and functional heterogeneity of the human proteome, breaking the simple one-gene-one-protein paradigm [4,5,6]. Accumulated evidence demonstrates that the approximately 20,000 protein-coding human genes are capable of generating millions of structurally and functionally distinct proteoforms predominantly driven by diverse PTM events [7,8]. Beyond proteomic diversification, PTMs also dynamically calibrate sophisticated intracellular and intercellular signaling cascades, endowing cells with the capacity to rapidly sense and adapt to fluctuating internal and external microenvironments, which is essential for the maintenance of cellular and organismal physiological homeostasis [9,10,11]. To date, more than 400 unique PTM subtypes have been discovered and validated in eukaryotic organisms, and the inventory of PTMs continues to expand with the continuous innovation of high-throughput proteomic detection techniques [6,12,13]. Despite such extensive diversity, protein tyrosine sulfation (PTS, Tyr-O-SO_3_) has long remained an underappreciated PTM. Initially identified in fibrinopeptide B in 1954, PTS was historically regarded as a rare modification with limited biological significance [14,15]. Fortunately, recent advances in biological detection technologies have recently overturned this cognition, definitively revealing the widespread distribution and functional importance of PTS, as well as its indispensable roles in multiple physiological and pathological processes.

Mechanistically, PTS refers to the sulfation modification occurring at tyrosine residues within proteins or peptides, involving the covalent attachment of the sulfate to the hydroxyl group of the tyrosine residues [16,17,18]. Reports of tyrosine sulfation across both animal and plant kingdoms establish it as a PTM common to eukaryotic proteins. For instance, tyrosine sulfation of certain plant peptides has been demonstrated to be a key regulator in plant growth and development processes [19,20,21,22,23]. Consistent with the canonical regulatory properties of protein PTMs, PTS is capable of modulating the secondary and tertiary structure, subcellular trafficking, and biological functionality of substrate proteins. Notably, a growing body of research indicates that the core molecular mechanism underlying PTS-mediated biological regulation lies in its potent capacity to modulate protein–protein interaction affinity and specificity, which endows modified proteins with unique functional characteristics and signal transduction activity [16,24]. Functionally, PTS participates in a broad spectrum of fundamental biological processes, including cell chemotaxis, blood coagulation, leukocyte trafficking, and immune antibody regulation [25,26,27]. More importantly, aberrant PTS modification is tightly linked to the initiation and progression of multiple human diseases, particularly infectious diseases and malignancies [26,28,29]. Given these functional implications, PTS has emerged as a novel and promising therapeutic target for human disease intervention.

## 2. Mechanism of Protein Tyrosine Sulfation

Sulfation (also termed sulfonation) is a widespread biochemical reaction in nature, encompassing plants, animals, and microorganisms [30,31]. It modifies a broad spectrum of biological substrates, such as hormones, neurotransmitters, carbohydrates, and proteins, to regulate multiple critical biological processes, including the blood coagulation cascade and connective tissue formation [30,32,33]. In the context of protein modification, sulfation predominantly occurs on specific tyrosine residues, and this unique PTM pattern is referred to as tyrosine sulfation (also known as tyrosine sulfonation) [34].

**Figure 1:**
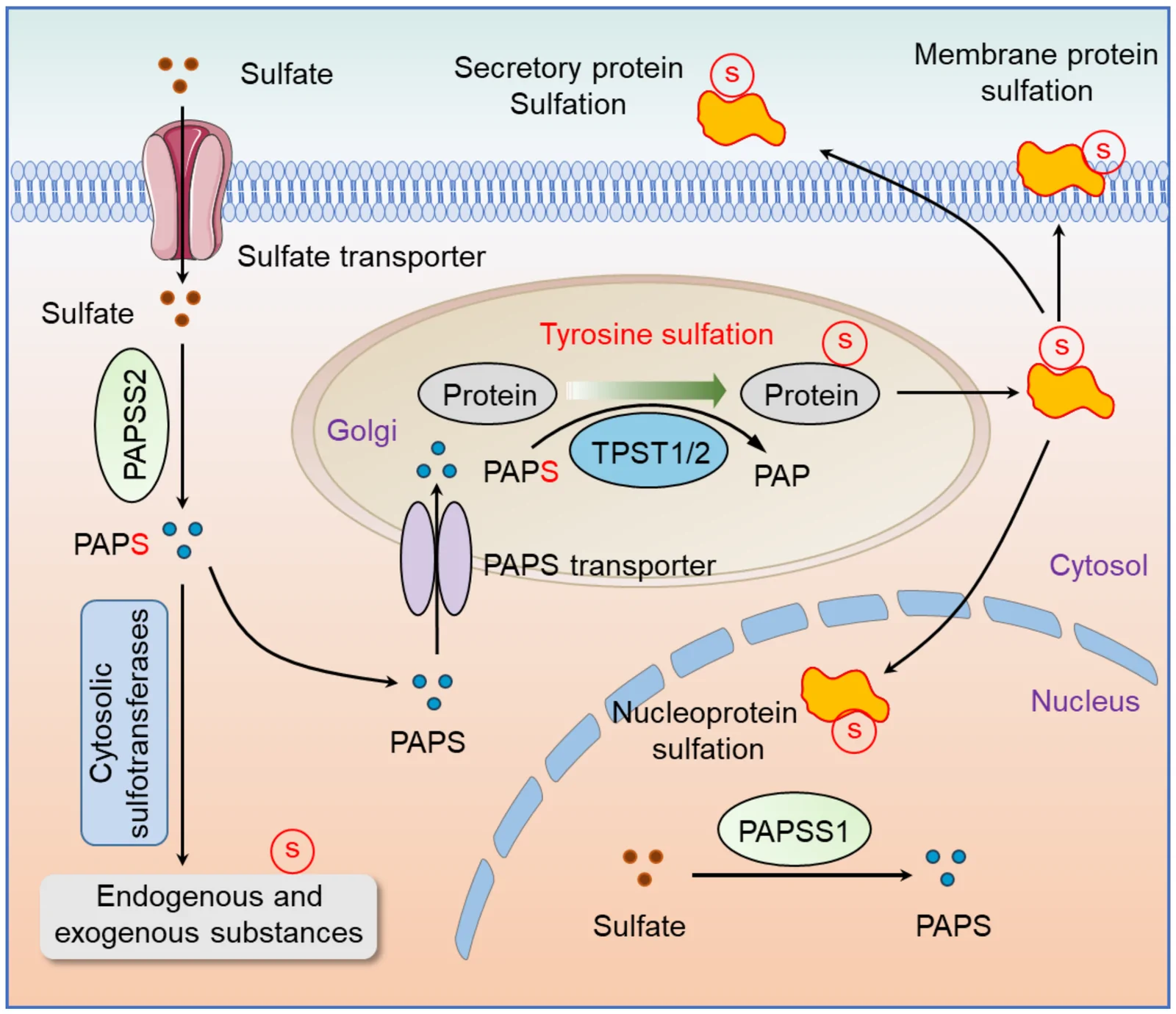
Schematic illustration of canonical PTS. The drawing depicts the process of canonical tyrosine sulfation. In mammalian cells, inorganic sulfate is converted into the universal sulfation donor PAPS via catalytic reactions mediated by PAPSS isoforms. In the nucleus, PAPSS1 catalyzes the conversion of inorganic sulfates to PAPS, while PAPSS2 performs the same reaction in the cytoplasm. Cytosolic PAPS serves as a substrate for cytosolic sulfotransferases, which modify endogenous molecules (e.g., hormones and neurotransmitters) and exogenous compounds (e.g., drugs). Cytosolic PAPS is also transported into the Golgi apparatus via the transporters SLC35B2 and SLC35B3, where tyrosine sulfation occurs. The newly sulfated proteins are subsequently trafficked to their subcellular or extracellular destinations to exert diverse biological functions. This schematic represents the canonical Golgi PTS pathway and does not depict proposed non-canonical cytoplasmic PTS events such as histone H3 sulfation.

The entire PTS catalytic cascade relies on the coordinated operation of multiple functional enzymes and specific solute transporters, constituting a sophisticated and tightly regulated molecular pathway (Figure 1). Given the high polarity and charge properties of inorganic sulfate, extracellular sulfate ions cannot penetrate the lipid bilayer via passive diffusion. Instead, specific plasma membrane transporters, mainly including SLC26A1 and SLC26A2, mediate the intracellular uptake of exogenous sulfate for subsequent sulfation reactions [35,36,37]. After entering the cytoplasm, inorganic sulfate requires enzymatic activation to form high-energy sulfate donors, which is achieved via two consecutive catalytic reactions mediated by PAPS synthase (PAPSS) [38,39,40]. Briefly, the first step involves the substitution of a phosphate group in adenosine triphosphate (ATP) by a sulfate group to generate adenosine-5′-phosphosulfate (APS), a key intermediate metabolite. The subsequent phosphorylation of APS produces the final activated sulfate donor, 3′-phosphoadenosine-5′-phosphosulfate (PAPS). Collectively, the synthesis of APS and PAPS represents a key step in the sulfation process. In humans, PAPS synthesis involves two reactions: ATP sulfurylase converts inorganic sulfate to APS, which is then phosphorylated by APS kinase to generate PAPS [26,28,39]. Notably, this dual-catalytic system exhibits distinct evolutionary divergence across species. In prokaryotes, fungi and plants, ATP sulfurylase and APS kinase exist as independent enzymes encoded by discrete genes, whereas animal genomes encode a single bifunctional PAPSS protein with dual catalytic activities [41,42]. To date, two human PAPSS paralogs, PAPSS1 and PAPSS2, have been successfully identified and characterized [43,44]. Although both isoforms display ubiquitous expression in human tissues, they differ markedly in subcellular localization and catalytic efficiency. Specifically, PAPSS1 predominantly localizes to the nucleus, while PAPSS2 is enriched in the cytoplasm, thereby partitioning PAPS synthesis into distinct subcellular compartments and supporting spatially specific sulfation modification [45,46,47]. Both APS and PAPS possess high-energy phosphor-sulfate anhydride bonds, which endow them with potent sulfuryl group donor activity to fuel downstream sulfation reactions [39]. Of note, sulfate utilization patterns vary drastically among species. Phototrophic bacteria, algae and partial plant species employ APS for assimilatory sulfate reduction to synthesize sulfur-containing amino acids (cysteine and methionine), while chemotrophic bacteria, fungi and higher plants prefer PAPS-dependent metabolic pathways. Unlike lower organisms, humans and other animals lack a complete sulfate reduction system, rendering methionine an essential amino acid that must be obtained from dietary intake [30,48]. Following PAPS biosynthesis, the sulfotransferase family is responsible for transferring the active sulfate group from PAPS to various target biomolecules [49,50]. Based on substrate specificity and subcellular localization, mammalian sulfotransferases are categorized into three major subfamilies: tyrosylprotein sulfotransferases (TPSTs), cytosolic sulfotransferases (SULTs), and carbohydrate sulfotransferases [51]. Among these subfamilies, TPSTs, consisting of two well-characterized isoforms (TPST1 and TPST2), exclusively mediate the sulfation of tyrosine residues on proteins and peptides, thus serving as the core catalytic enzymes for PTS [52,53,54]. Given the focus of this review on protein tyrosine sulfation, TPSTs will be emphasized hereafter. TPSTs are typical Golgi-resident enzymes with a highly conserved sulfotransferase domain across species, highlighting the evolutionary stability and functional indispensability of PTS modification [55]. Significant advances have been made in the molecular cloning of both TPST isoforms, and no additional TPST members have been reported [56,57,58]. TPST1 and TPST2 were first discovered and characterized in the laboratory of Kevin L. Moore [59,60]. By generating gene knockout models, his group delineated the distinct physiological roles of TPST1 and TPST2. In particular, loss of TPST2 causes male infertility in mice [61]. Subsequent studies further revealed that TPST2-mediated sulfation participates in regulating the sperm surface proteome or membrane order, ultimately affecting male fertility [62]. Accumulating evidence has confirmed that TPST1 and TPST2 exhibit distinct substrate specificities, targeting distinct subsets of client proteins to mediate diverse PTS events, as verified by multiple in vitro catalytic assays [63]. As TPST is Golgi-resident, the cytosolic sulfate donor PAPS must first be transported into the Golgi lumen by specific PAPS transporters (SLC35B2 and SLC35B3) to enable tyrosine sulfation. Ultimately, TPST-dependent PTS modification remodels the conformational structure, surface charge distribution and biochemical properties of target proteins, which modulates protein stability, molecular interaction and subcellular trafficking, thereby participating in the regulation of multiple cellular activities [54,64,65,66].

Most currently identified sulfated proteins acquire PTS via TPST-mediated catalysis. However, atypical tyrosine sulfation has been described for a small subset of substrates, such as histone H3, although this observation remains controversial. Unlike canonical PTS, which takes place in the Golgi apparatus and depends on TPST enzymes, histone H3 undergoes tyrosine sulfation at Y99 (H3Y99sulf) within the cytoplasm. This reaction is reportedly catalyzed by cytosolic sulfotransferases SULT1B1 and GAL3ST1 [67,68]. This non-canonical pathway remains poorly characterized and mechanistically distinct from TPST-dependent PTS. Specifically, how the relevant substrates gain access to cytoplasmic sulfotransferases and many core mechanistic details of this histone sulfation event remain incompletely resolved.

## 3. Detection of Protein Tyrosine Sulfation

Tyrosine is a non-essential amino acid that is either exogenously acquired from dietary intake or endogenously synthesized in the human liver via the hydroxylation of the essential amino acid phenylalanine [69,70]. Both tyrosine and its metabolic precursor phenylalanine serve as initial precursors for the biosynthesis of neurotransmitters or hormones in animals [71,72,73]. Once incorporated into peptide chains, the phenolic hydroxyl group of tyrosine residues is susceptible to diverse post-translational modifications, including nitration, phosphorylation, and sulfation, each of which confers distinct structural and functional properties to substrate proteins [74,75]. Notably, tyrosine nitration predominantly occurs through non-enzymatic oxidative reactions, whereas tyrosine phosphorylation and PTS are strictly catalyzed by specific modifying enzymes, representing precisely regulated enzymatic PTMs [71,74]. Phosphate and sulfate groups are not only core participants in intracellular energy metabolism but also serve as negatively charged functional moieties that alter the surface electrostatic properties of target biomacromolecules [76]. Despite their highly analogous geometric configuration and molecular size, subtle discrepancies in the valence electron distribution of their central phosphorus and sulfur atoms lead to divergent characteristics in charge distribution, metal ion binding affinity, and molecular solubility. These intrinsic chemical differences ultimately drive the distinct biological functions of the two modifications in cellular regulatory networks [76]. To date, abundant functional studies have clearly delineated their functional divergence: tyrosine phosphorylation acts as a core signaling hub to orchestrate cellular transduction cascades and enzymatic activity, while PTS primarily modulates protein conformational changes and protein–protein interaction specificity [24,77,78]. Of particular note, no mammalian desulfation enzyme has been identified so far [74,79]. Although PTS lacks dedicated enzymatic reversal under physiological conditions, it is not chemically irreversible. The sulfate group in PTS is quite stable under neutral and alkaline pH conditions but highly labile in acidic environments, which raises the possibility of non-enzymatic desulfation within acidic intracellular compartments such as lysosomes [74]. While tyrosine sulfation and phosphorylation introduce chemically similar functional groups (-SO_3_H and -PO_3_H_2_, respectively), PTS differs fundamentally from tyrosine phosphorylation: phosphorylation is dynamically turned over by phosphatases, whereas PTS lacks a comparable enzymatic removal system. Furthermore, research progress for these PTMs has been uneven. Advances in analytical methodologies have propelled rapid and extensive study of protein phosphorylation. In contrast, PTS research continues to encounter challenges stemming from its low intracellular abundance, inherent instability, and a scarcity of effective enrichment strategies [80].

Elucidating the biological functions of PTS first requires the identification of sulfated proteins and their precise modification sites. Currently, the most straightforward and efficient preliminary screening method involves using bioinformatics databases to assess the likelihood of tyrosine sulfation in target proteins and identify potential modification sites. Frequently employed web-accessible prediction tools are Sulfinator, SulfoSite, and PredSulSite [26,81,82]. Nevertheless, due to the limited scale and incomplete coverage of existing PTS databases, in silico prediction inevitably generates substantial false-positive results. Therefore, all candidate PTS substrates and modification sites require experimental validation to confirm their authenticity.

Currently, isotope labeling remains the most classical and widely recognized experimental approach for PTS detection. In this approach, radiolabeled PAPS with ^35^S serves as the sulfate group donor. Under the catalysis of TPST, the ^35^S-labeled sulfate group is transferred to tyrosine residues of protein or peptide substrates, thereby achieving tyrosine sulfation. After incorporation of ^35^S, the radioactive signal can be detected by autoradiography or scintillation counting, reflecting the PTS level of the target protein. The specific experimental procedure is as follows: First, ^35^S-labeled sulfate is added to cell culture media or injected into an animal’s tissue of interest. The labeled sulfate is subsequently metabolized to generate ^35^S-PAPS. Subsequently, TPST catalyzes the transfer of the ^35^S-labeled sulfate group to tyrosine residues of the target protein, completing the modification process. Finally, the modified products can be analyzed using thin-layer chromatography (TLC). Alternatively, incorporated ^35^S can be directly measured with a scintillation counter or visualized by autoradiography after SDS-polyacrylamide gel electrophoresis (SDS-PAGE) [15,58,83,84]. This pioneering method has laid the foundation for early PTS research, enabling the identification and verification of most classic sulfated proteins. However, it possesses several unavoidable limitations. First, this approach only detects the global PTS modification level of intact proteins, failing to pinpoint exact modified tyrosine residues. Second, the application of radioactive substances entails complex operational procedures, high experimental risks, and strict radiation safety management standards, which greatly restrict its widespread application.

In recent years, the development of a high-specificity commercial mouse monoclonal antibody targeting sulfated tyrosine has provided a powerful and convenient immunoassay tool for PTS qualitative and quantitative detection. Its screening antigens were KAKISDP-DY(SO_3_)MTGYMDAC and KDKKYATEY(SO_3_)-EYLDYDFC. This antibody specifically recognizes sulfated tyrosine residues on various proteins, does not recognize phosphorylated or unmodified tyrosine residues, and its recognition is independent of the surrounding amino acid sequence [85]. Combined with conventional experimental platforms, including Western blotting, enzyme-linked immunosorbent assay (ELISA), and flow cytometry, this antibody enables systematic quantification of PTS levels in diverse cell and tissue samples. Furthermore, it supports the affinity enrichment of unknown sulfated proteins and facilitates the exploration of differential substrate specificity between TPST1 and TPST2, greatly advancing functional and mechanistic PTS research.

Mass spectrometry (MS) is the most authoritative and high-resolution technique for large-scale PTM identification and site localization, and this holds true for the detection of PTS modification as well. Most common PTMs, such as glycosylation, ubiquitination, phosphorylation, acetylation, and methylation, exhibit robust stability under standard MS detection conditions, enabling efficient identification and quantitative analysis. PTS, however, is uniquely challenging for MS detection due to its inherent acid lability. Since protein separation and analysis procedures are typically conducted in acidic environments, the sulfate group on PTS is highly susceptible to cleavage under these conditions, resulting in severe degradation of PTS signals and frequent false-negative detection outcomes [26,86,87]. Compounding this issue, the molecular masses of the sulfate group (-SO_3_, 79.9568 Da) and phosphate group (-PO_3_H_2_, 79.9663 Da) are extremely similar, with a tiny mass difference of merely 0.0095 Da, making it technically difficult to precisely distinguish these two modifications via conventional MS analysis [88,89]. In addition, low-abundance PTMs, including PTS, are frequently lost during bulk MS measurements [90]. Furthermore, MS-based PTS detection requires sophisticated operational proficiency and high-end precision instruments, which greatly increases experimental costs and limits the large-scale popularization and systematic application of PTS proteome profiling. Collectively, these multi-layered technical barriers severely hinder the in-depth systematic study of PTS biological functions and regulatory mechanisms. Nevertheless, pioneering work has demonstrated that MALDI-TOF-MS analysis of sulfotyrosine-containing peptides, performed with α-cyano-4-hydroxycinnamic acid as the matrix in the negative-linear, delayed-extraction mode, could partially mitigate this acid-dependent analytical obstacle [91,92]. Building on these early efforts, modern sulfoproteomic workflows integrate sulfopeptide enrichment, pH-regulated sample preparation and customized LC–MS acquisition to stabilize acid-labile sulfate groups and distinguish sulfotyrosine from near-isobaric phosphotyrosine, enabling large-scale profiling of endogenous PTS sites in complex samples [14,53,91,92,93]. In parallel, chemoenzymatic and chemical synthesis methods produce site-specific, homogeneously sulfated peptides and proteins. In contrast to cellular TPST expression, which generates heterogeneous mixtures of modified and unmodified protein isoforms, these synthetic constructs have precisely defined stoichiometry. They act as reference standards for MS validation and are critical reagents for rescue assays to investigate PTS-dependent protein functions [93].

Following the identification of PTS-modified proteins, further functional characterization requires comparative analysis between sulfated and non-sulfated protein statuses, which faces multiple technical hurdles. The first challenge is efficient production. Although the PTS-catalyzing enzyme TPST can generate sulfated proteins, it is only expressed in organisms and sulfates multiple substrates, often yielding heterogeneous mixtures [15]. Second, complex modification patterns increase experimental difficulty. Most PTS substrates contain multiple putative tyrosine sulfation sites, which can be fully or partially modified in a context-dependent manner. Moreover, modification at a single site may exert synergistic or antagonistic effects on adjacent sites, rendering it challenging to dissect the independent biological function of individual PTS sites. Site-directed mutagenesis is therefore indispensable for functional verification, further complicating experimental design and operation [94]. Given the structural similarity between tyrosine and phenylalanine, as well as the resistance of phenylalanine to sulfation mutation, tyrosine-to-phenylalanine (Y-to-F) site mutagenesis has become the most classic and universal strategy to specifically ablate endogenous PTS modification for functional phenotypic comparison [93,95]. Third, the lack of specific pharmacological tools restricts functional exploration. To date, no highly specific small-molecule inhibitors or antagonists targeting PTS or TPSTs have been developed, hindering the precise modulation of PTS levels under physiological and pathological conditions and limiting the in-depth validation of PTS biological functions [96]. Importantly, neither broad inhibition of sulfation nor Y-to-F substitution alone is sufficient to establish a sulfate-specific mechanism. Global sulfation inhibition simultaneously suppresses sulfation across many cellular substrates, so any observed phenotypic change cannot be unambiguously attributed to a single tyrosine sulfation event. Similarly, Y-to-F mutagenesis eliminates sulfation at that site, but this amino acid substitution may alter protein folding, local surface charge, or protein–protein interactions independent of sulfate modification itself. Consequently, these approaches only provide preliminary correlative evidence. Definitive proof for sulfate-specific function requires orthogonal rescue experiments using chemically synthesized, homogeneously sulfated peptides or proteins to restore the phenotype.

## 4. Roles of Protein Tyrosine Sulfation

With the continuous advancement of detection techniques and functional validation strategies, the biological significance of PTS has been progressively uncovered in recent years. PTS is recognized as a highly specific and conserved PTM that predominantly takes place in the Golgi apparatus, with secreted proteins and transmembrane receptors serving as its canonical substrates [97,98]. Nevertheless, this traditional understanding has been substantially updated by emerging studies identifying PTS modification on multiple intracellular proteins, such as the tumor suppressor p53 and epigenetic core component histone H3. These discoveries greatly broaden the substrate spectrum of PTS and extend its regulatory scope beyond membrane and extracellular proteins [67,99]. To date, most known functions of PTS are linked to regulating protein–protein interactions, likely due to the large surface area, charged nature, and hydrogen-bonding capacity of the sulfate group [100,101,102]. Owing to these features, PTS participates in a wide range of fundamental physiological processes, including cell adhesion, leukocyte trafficking, inflammatory response activation, and hormone signal modulation [25,71,103]. A well-characterized example is the PTS modification of chemokine receptors, including CCR8, CCR5, CCR2, CX3CR1, CXCR4, and CXCR3, whose sulfation status is critical for high-affinity binding to their cognate chemokine ligands [104,105,106]. Taking the chemokine receptor CXCR4 as an example, this protein possesses multiple tyrosine sulfation sites (Y7, Y12, and Y21). Sulfation at these sites enhances its affinity for its ligand CXCL12, thereby regulating cell migration and chemotaxis [107]. Given its functional relevance, dysregulated PTS is increasingly implicated in disease. Recent studies have linked PTS to major human diseases, including infectious diseases (e.g., AIDS and malaria), cancers (e.g., nasopharyngeal and pancreatic), and immune-mediated conditions (e.g., allergies and rheumatoid arthritis) [71,84,93,108]. Many of these findings are derived from cellular or animal studies. Human associative evidence is limited, and robust clinical validation is not yet available. As such, PTS has emerged as a novel entry point for disease research, following classical and emerging protein modifications like phosphorylation, acetylation, SUMOylation, and palmitoylation, and holds promise as a potential therapeutic target for human diseases. In addition, multiple studies have reported associations between genes involved in the PTS modification process (including SLC35B2, SLC35B3, TPST1, TPST2, PAPSS1, PAPSS2, SLC26A1, and SLC26A2) and diseases. Although its mechanism of action is not yet fully understood, its underlying mechanism may also be related to the sulfation modification of specific downstream proteins regulated by these genes. Overall, PTS possesses important biological functions, although many mysteries surrounding this modification remain unresolved. In this review, we systematically summarize the currently identified sulfated proteins and classify them according to their subcellular localization. Furthermore, we discuss the potential roles of PTS in physiological homeostasis and disease pathogenesis based on its intrinsic biochemical properties.

### 4.1. Membrane Proteins

#### 4.1.1. Chemokine Receptor

Chemokines, also known as chemotactic cytokines, are a group of small proteins (8–10 kDa) induced by inflammatory cytokines, growth factors, and pathogenic stimuli and possess the function of directionally regulating cell migration [109,110,111]. As secreted proteins, chemokines exert their biological effects by binding to specific G-protein-coupled transmembrane receptors (GPCRs) on the cell surface and activating intracellular signaling pathways [112,113,114]. Based on the position of the two conserved N-terminal cysteine residues, chemokines are classified into four subfamilies: C, CC, CXC, and CX3C [115,116]. Accordingly, their receptors are named after the chemokine subfamily they bind, designated as XCR, CCR, CXCR, and CX3CR, respectively [117,118]. The chemokine-receptor axis plays critical roles in various physiological and pathological processes, including early embryonic development, immune surveillance, and inflammatory responses [119,120]. For instance, during infectious diseases, chemokines induce the migration of antigen-presenting cells, effector T cells, and B cells to sites of infection or lymphoid organs, thereby enhancing pathogen clearance. In autoimmune diseases, however, chemokines may promote the infiltration of self-reactive T and B cells into target organs, exacerbating tissue damage [121,122]. Therefore, functional imbalance of the chemokine-receptor signaling axis is tightly correlated with the occurrence and progression of multiple human disorders, rendering this axis a promising target for disease intervention.

**Figure 2:**
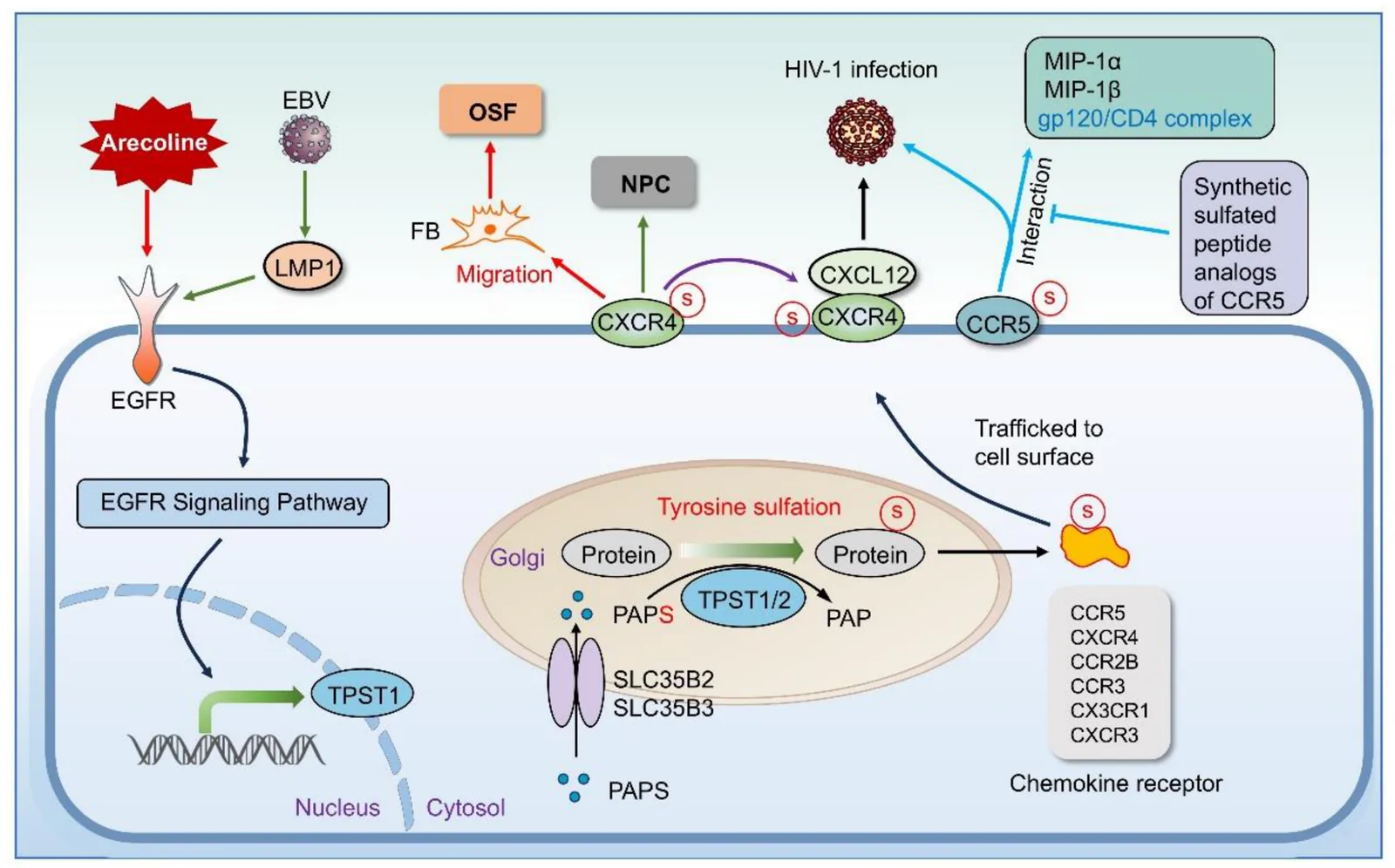
Regulation of chemokine receptor function by PTS. Several chemokine receptors (e.g., CCR5, CXCR4, CCR2B, CCR3, CX3CR1, CXCR3) undergo PTS, which regulates their functions. For CCR5, sulfation enhances binding to MIP-1α, MIP-1β, and HIV-1 gp120/CD4, thereby promoting HIV-1 infection. Notably, synthetic sulfated CCR5-derived peptides can block this entry. In contrast to CCR5, CXCR4 sulfation increases its binding affinity for CXCL12 and stabilizes the receptor–ligand complex; this modification only modestly facilitates HIV-1 infection. Beyond viral infection, PTS-modified chemokine receptors are also involved in other diseases. In OSF, arecoline induces CXCR4 sulfation via TPST1, enhancing fibroblast migration. In NPC, the Epstein–Barr virus (EBV)-encoded LMP1 upregulates TPST1 through the LMP1/EGFR axis, increasing CXCR4 sulfation and driving malignancy, although the precise mechanisms remain unclear.

Recent studies have revealed that several chemokine receptors (including CCR5, CXCR4, CCR2B, CCR3, CX3CR1 and CXCR3) undergo tyrosine sulfation (PTS), which is crucial for regulating their functions (Figure 2) [71,104,123]. PTS modification predominantly occurs on the extracellular N-terminal domain of these receptors, a key region responsible for ligand recognition and binding, thus endowing PTS with potent regulatory effects on receptor–ligand interaction and downstream signaling activation. Taking CCR5 as an example, it has been identified to possess four sulfation sites (Y3, Y10, Y14, and Y15) [124,125]. Its sulfation status facilitates the binding of CCR5 to MIP-1α and MIP-1β, and more importantly, facilitates the specific assembly of the HIV-1 gp120/CD4/CCR5 complex. This modification-dependent molecular interaction is a prerequisite for efficient HIV-1 viral attachment, membrane fusion, and subsequent invasion of host immune cells, thereby promoting viral infection and replication (Figure 2) [65,126,127,128]. Given the important role of CCR5 sulfation in HIV infection, researchers have sought to develop antiviral strategies targeting this modification. Based on the chemical characteristics of PTS and its mechanism of action, the feasibility and efficacy of such therapeutic strategies are being progressively validated. Notably, synthetic sulfated peptide analogs of CCR5 can competitively bind to HIV-1 gp120, effectively blocking the interaction between viral proteins and host CCR5 and thereby inhibiting viral entry into target cells [129,130,131]. In addition, while CXCR4 sulfation contributes to HIV-1 infection, its effect is substantially weaker than that of CCR5 sulfation (Figure 2) [132,133]. CXCR4 contains multiple PTS sites (Y7, Y12, and Y21), and its sulfation enhances its binding affinity for its cognate ligand CXCL12, a well-established function. This conformational change may indirectly generate a receptor state permissive for recognition by HIV-1 gp120, but CXCR4 sulfation is not an essential determinant for viral entry. The underlying mechanism primarily involves the formation of hydrogen bonds by PTS on the dimer surface, which alters the surface conformation and increases electrostatic binding energy, thereby stabilizing the complex structure [107,134]. In summary, sulfation of CXCR4 enhances its binding affinity for the natural ligand CXCL12, thereby creating a more favorable receptor environment for HIV infection. However, this promoting effect is far less indispensable than CCR5 sulfation during HIV-1 infection. This reveals that HIV-1 employs distinct molecular strategies when hijacking different coreceptors.

In addition to their involvement in viral infections, PTS-modified chemokine receptors also participate in the pathogenesis of other diseases, including mucosal fibrotic lesions and malignant tumors. Oral submucous fibrosis (OSF), a condition closely associated with areca nut chewing, is an irreversible oral mucosal disease with malignant potential [135,136]. Mechanistically, arecoline promotes the sulfation of CXCR4 via TPST1, thereby enhancing the migration capacity of fibroblasts (FBs) and ultimately facilitating the progression of OSF (Figure 2) [137]. Furthermore, CXCR4 sulfation is also closely linked to nasopharyngeal carcinoma metastasis. Latent membrane protein 1 (LMP1), an oncoprotein encoded by the Epstein–Barr virus (EBV), promotes TPST1 transcription in a dose-dependent manner through the LMP1/EGFR/TPST1 signaling axis, upregulating CXCR4 sulfation levels and thereby driving the malignant progression of nasopharyngeal carcinoma (NPC) (Figure 2) [84]. Nevertheless, the CXCR4 sulfation sites and the downstream signaling cascades in NPC remain to be fully elucidated, which requires further in-depth mechanistic exploration.

#### 4.1.2. PSGL-1

**Figure 3:**
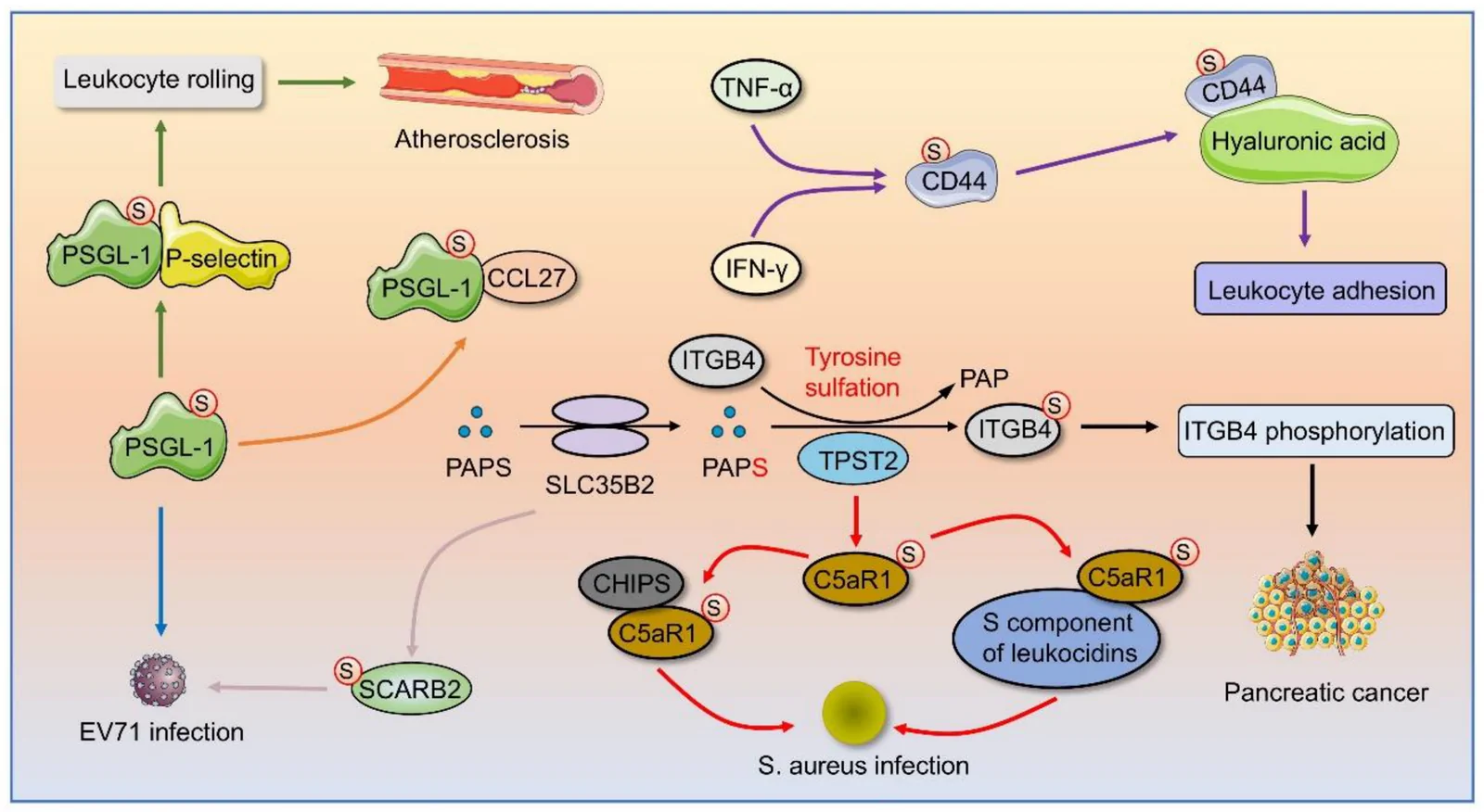
Tyrosine sulfation of membrane proteins in inflammation, infection, and cancer. N-terminal tyrosine sulfation of PSGL-1 strengthens its interactions with P-selectin and CCL27. The strengthened PSGL-1/P-selectin binding further promotes leukocyte rolling, a key event underlying atherosclerotic progression. This modification also facilitates EV71 virus entry into leukocytes, whereas removal of sulfate groups or tyrosine mutagenesis reduces infection. For C5aR1, sulfation strengthens its binding to the *Staphylococcus aureus* immune evasion protein CHIPS. Moreover, TPST2-mediated sulfation of C5aR1 enhances its interaction with the S component of leukocidins, thereby promoting bacterial pathogenesis. In the case of CD44, pro-inflammatory cytokines (TNF-α, IFN-γ) induce CD44 sulfation, converting it to an active form that binds hyaluronic acid and endothelial cells, thus regulating leukocyte adhesion during inflammation. As an EV71 entry receptor, SCARB2 is sulfated at multiple tyrosine sites mediated by SLC35B2, which enhances viral binding and infection. In pancreatic cancer, the SLC35B2-TPST2 axis sulfates ITGB4, increasing its stability and phosphorylation and consequently driving cancer progression.

P-selectin glycoprotein ligand-1 (PSGL-1) is a vital transmembrane glycoprotein involved in the initiation of inflammatory responses [138,139]. Expressed on the surface of most leukocytes, it serves as the primary ligand for all three selectins (P-selectin, E-selectin, and L-selectin) [140,141]. Under basal physiological conditions, PSGL-1 remains in a low-affinity state to maintain vascular homeostasis. Upon inflammatory activation, PSGL-1 exhibits high binding affinity for endothelial selectins. The specific PSGL-1–selectin interaction mediates the rolling, firm adhesion, and migration of leukocytes and platelets along vascular endothelial layers, which constitutes the initial and rate-limiting step of local inflammatory responses and immune cell recruitment [97,142,143]. Owing to its indispensable role in governing inflammatory cell trafficking, dysfunctional PSGL-1 signaling is tightly implicated in the pathogenesis of multiple human disorders, particularly atherosclerotic vascular diseases and diverse viral infectious diseases [144,145,146]. Notably, the sulfation modification of PSGL-1 plays a key role in regulating its function. Current studies indicate that the amino-terminal region of PSGL-1 contains three potential tyrosine sulfation sites, and sulfation at these residues critically modulates the binding between PSGL-1 and P-selectin [147,148]. For instance, in the context of atherosclerosis, PTS-modified PSGL-1 exhibits significantly enhanced affinity for endothelial P-selectin. This strengthened molecular interaction promotes excessive monocyte recruitment and persistent inflammatory infiltration at atherosclerotic lesion sites, accelerates vascular endothelial injury and lipid plaque deposition, and ultimately drives the initiation and progressive exacerbation of atherosclerotic lesions (Figure 3) [149]. Beyond its role in the interaction between PSGL-1 and P-selectin, tyrosine sulfation also influences the binding of PSGL-1 to the chemokine CCL27. Studies have shown that treatment with arylsulfatase, which removes sulfate groups from tyrosine residues on PSGL-1, reduces its sulfation level and subsequently weakens its binding capacity to CCL27. Similarly, sulfation inhibitors inhibit this interaction. Furthermore, mutating the amino-terminal tyrosine residues of PSGL-1 to phenylalanine also significantly diminishes binding. Collectively, these findings indicate that tyrosine sulfation plays an important role in regulating the interaction between PSGL-1 and CCL27 (Figure 3) [150]. In addition to its involvement in chemokine binding, tyrosine sulfation of PSGL-1 also plays a crucial role in viral infection. Human enterovirus 71 (EV71), a member of the Picornaviridae family, is a major pathogen of hand, foot, and mouth disease in infants and young children [151,152]. PTS modification of PSGL-1 has been identified as an essential host factor facilitating EV71 attachment and invasion into leukocytes. Specifically, genetic ablation of PTS sites via site-directed mutagenesis or pharmacological blockade of sulfation with sodium chlorate efficiently reduces endogenous PSGL-1 sulfation levels. Reduced PTS modification significantly weakens the binding ability of PSGL-1 to EV71 viral particles, thereby suppressing viral attachment, host cell entry, and subsequent intracellular viral replication. These findings highlight the potential of targeting PSGL-1 sulfation as a novel preventive and therapeutic strategy for EV71-associated infectious diseases (Figure 3) [153].

#### 4.1.3. C5aR1

The complement system serves as a core constituent of innate immunity, acting as a first-line defensive barrier against invasive pathogens and participating in the modulation of inflammatory homeostasis and adaptive immune responses [154,155]. As one of the most potent bioactive effector fragments generated during complement cascade activation, C5a exerts its biological functions by specifically engaging two distinct membrane receptors, namely C5aR1 and C5aR2, thereby initiating divergent downstream signaling cascades [156]. C5aR1, also known as C5a anaphylatoxin chemotactic receptor 1 (C5a-R or CD88), is a typical G protein-coupled receptor (GPCR) widely expressed on the surface of immune cells such as neutrophils, monocytes, and macrophages [157,158]. Functionally, upon complement activation and C5a generation, C5a binds to C5aR1 and triggers downstream G protein signaling pathways, leading to chemotactic migration, degranulation, respiratory burst, and cytokine release, thereby mediating inflammatory responses and immune defense [157,159,160]. In contrast, C5aR2 represents an atypical receptor that lacks G protein coupling capability and predominantly functions through recruiting β-arrestin-dependent signaling pathways, thereby exerting distinct or even antagonistic regulatory effects on immune responses [161,162]. Dysregulation of the C5a-C5aR1 signaling axis is closely associated with various inflammatory and immune-mediated diseases [163,164].

Notably, sulfation of C5aR1 has been implicated in *Staphylococcus aureus* (*S. aureus*) infection [29,165,166]. *S. aureus* is a predominant opportunistic pathogen responsible for a wide range of pulmonary and systemic infectious diseases. Notably, the widespread prevalence of methicillin-resistant *S. aureus* (MRSA) has severely compromised clinical therapeutic efficacy and poses an escalating global public health challenge [167,168]. To survive and propagate within host tissues, *S. aureus* has evolved sophisticated immune evasion strategies. Staphylococcal chemotaxis inhibitory protein (CHIPS), a key virulence factor secreted by *S. aureus*, functions as a high-affinity natural inhibitor of C5aR1. CHIPS binds to C5aR1, effectively blocking C5a-induced immune cell activation and suppressing the recruitment of neutrophils and monocytes to infection sites, which facilitates bacterial immune escape [169,170,171,172]. Mechanistically, two conserved tyrosine residues (Y11 and Y14) located within the N-terminal domain of C5aR1 undergo specific PTS modification. This sulfation pattern markedly enhances the electrostatic interaction and structural binding stability between C5aR1 and CHIPS, which is indispensable for the high-affinity binding of CHIPS to host receptors and subsequent immune suppression (Figure 3) [165,166]. Another major determinant of *S. aureus* disease severity is the release of bicomponent pore-forming toxins (PFTs), which cause host cell lysis by binding to specific receptors on the target cell surface [29,173]. A recent study has revealed a key molecular mechanism underlying this process: in phagocytes, TPST2 promotes the sulfation of C5aR1, which in turn enhances the interaction between host cells and the S component of leukocidins. This strengthened interaction ultimately facilitates the progression of *S. aureus* infection, thereby contributing to increased disease severity (Figure 3) [29]. Collectively, these findings demonstrate that C5aR1 sulfation serves as a double-edged regulator during *S. aureus* infection, which not only assists bacterial immune evasion but also amplifies toxin-mediated pathogenic damage.

#### 4.1.4. CD44

CD44 is a widely expressed cell adhesion molecule implicated in diverse biological processes, including lymphopoiesis, angiogenesis, wound healing, leukocyte extravasation during inflammation, and tumor progression [174,175]. In the context of inflammatory immune responses, CD44 facilitates the adhesion of leukocytes to vascular endothelial cells. Under resting conditions, CD44 on leukocytes exists in an inactive conformation that cannot bind its primary ligand, hyaluronic acid. However, upon appropriate stimulation, such as antigen recognition or cytokine exposure, CD44 undergoes a transition to an active state [176,177].

Notably, CD44 can undergo two distinct types of sulfation modifications: carbohydrate sulfation on its glycan chains and PTS within its core polypeptide [174,178,179,180]. The following discussion focuses specifically on CD44 polypeptide PTS and does not address glycan-associated carbohydrate sulfation. Studies have suggested that PTS modification acts as an important molecular switch governing the conformational activation and functional transformation of CD44. Classic pro-inflammatory cytokines, including TNF-α and IFN-γ, can effectively trigger CD44 sulfation, converting CD44 from its inactive to its active form. PTS endows it with robust binding capacity for both hyaluronic acid and vascular endothelial surface ligands. Functionally, PTS-dependent CD44 activation significantly enhances leukocyte-endothelial adhesion and transendothelial migration, thereby promoting massive leukocyte infiltration into inflamed tissues and amplifying local inflammatory responses (Figure 3). Consequently, modulating the tyrosine sulfation of CD44 may represent a potential strategy to regulate CD44-mediated leukocyte adhesion at sites of inflammation [174,179,180]. However, the precise tyrosine sulfation residues within CD44 and its downstream regulatory mechanisms remain to be fully validated.

#### 4.1.5. SCARB2

In addition, scavenger receptor class B member 2 (SCARB2), an EV71 virus entry protein, has the potential for PTS modification, and its sulfation is critical for viral replication [181]. As an indispensable functional receptor for EV71, SCARB2 not only mediates viral attachment and adsorption on the host cell surface but also facilitates subsequent intracellular viral uncoating and genome release, thereby determining the efficiency of EV71 infection and replication [182,183]. Mechanistically, the Golgi-resident PAPS transporter SLC35B2 has been identified as a key regulator of EV71 infection. SLC35B2 transports sufficient PAPS sulfate donors into the Golgi lumen to support efficient PTS catalysis, which promotes site-specific tyrosine sulfation of SCARB2. PTS modification significantly enhances the binding affinity between SCARB2 and EV71 viral particles, thereby markedly boosting viral attachment, internalization, and intracellular replication efficiency (Figure 3). Multiple tyrosine sulfation sites have been mapped as candidate residues within the SCARB2 protein sequence, including Y7, Y198, Y267, Y284, and Y410. Mutating these five tyrosine residues to phenylalanine did not abolish EV71 susceptibility, while mutation of all 20 tyrosine residue substitutions abrogated infection, indicating these five residues are not individually essential for infection. Nevertheless, the exact functionally relevant tyrosine-sulfated sites and spatiotemporal regulation of SCARB2 PTS remain unresolved. In addition, SLC35B2-mediated sulfation of heparan sulfate (HS) is also essential for EV71 infection. In summary, this study identifies SLC35B2 as a novel host factor governing EV71 cellular entry by regulating sulfation processes, including HS sulfation and protein tyrosine sulfation [181]. These findings substantially deepen the mechanistic understanding of host–virus interaction during EV71 infection, illustrate important roles of distinct sulfation events in facilitating viral invasion, and offer innovative targets and theoretical foundations for the development of novel anti-EV71 therapeutic strategies.

#### 4.1.6. ITGB4

Integrin beta4 (ITGB4), a member of the integrin family, functions as a mechanosensor that mediates bidirectional signaling between the intracellular and extracellular matrices [184]. Accumulating evidence indicates that ITGB4 is aberrantly overexpressed in multiple solid tumors, including lung, colon, and pancreatic cancers, and that its expression level correlates strongly with poor prognosis [185,186,187,188]. Focusing on pancreatic cancer, one study revealed that ITGB4 undergoes tyrosine sulfation, which is critical for its stability and function. This process is governed by a sulfation signaling axis consisting of SLC35B2 and TPST2. Activation of this axis enhances ITGB4 sulfation, thereby increasing its protein stability and phosphorylation, which in turn promotes pancreatic cancer progression. Consistently, knockdown of either SLC35B2 or TPST2, or pharmacological inhibition of tyrosine sulfation, effectively suppresses tumor progression (Figure 3). These findings underscore the pivotal role of the SLC35B2-TPST2-ITGB4 sulfation axis in pancreatic cancer malignancy. Targeted intervention against this PTS regulatory axis provides a novel therapeutic direction for precise targeted treatment of pancreatic cancer and other ITGB4-driven malignant tumors, possessing great potential for future clinical translation [108].

### 4.2. Nuclear Proteins

#### 4.2.1. Histone H3

**Figure 4:**
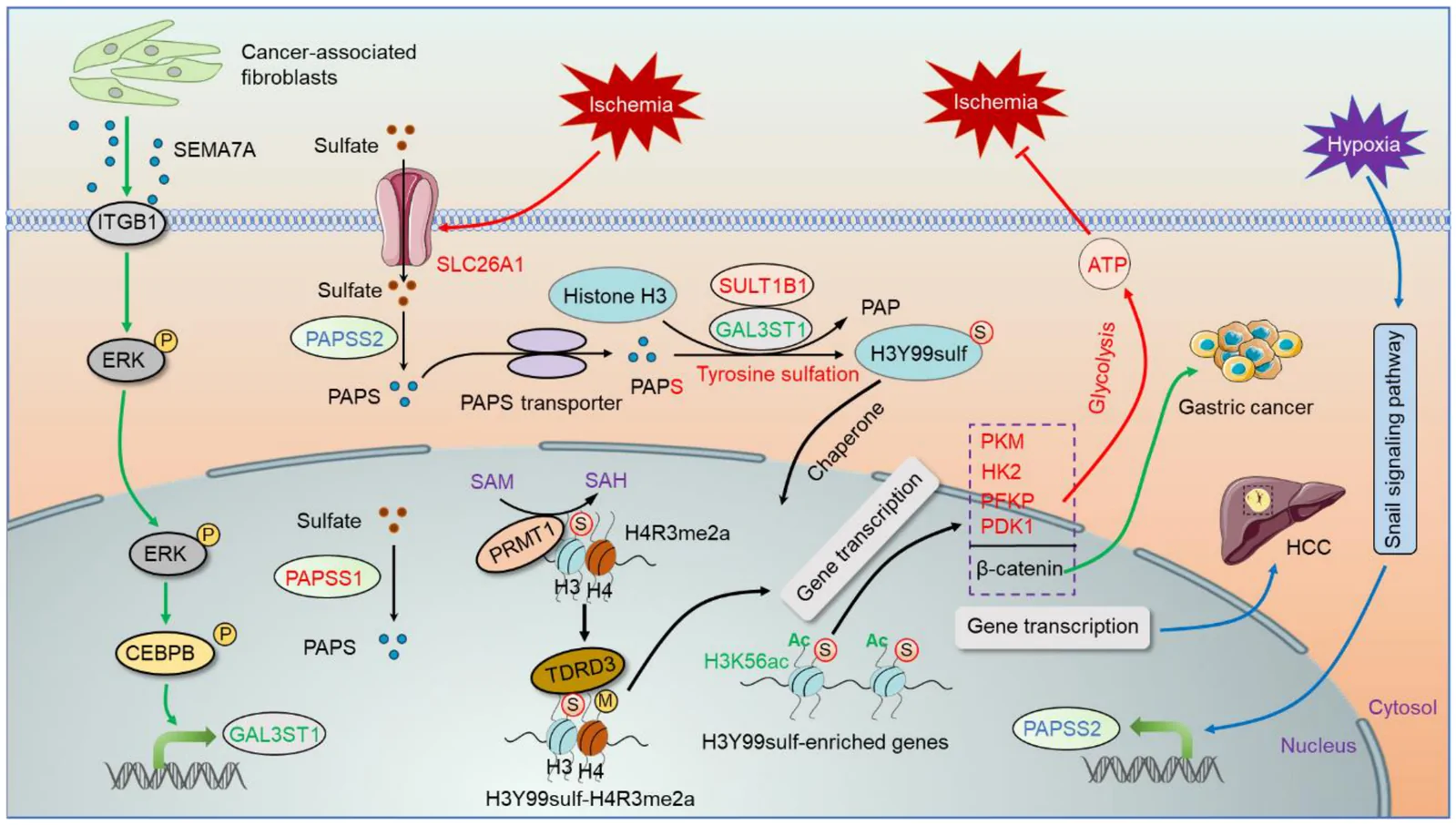
Proposed H3Y99sulf and its roles in transcriptional regulation and disease. Unlike classical Golgi-localized PTS, histone H3 has been reported to undergo sulfation at tyrosine 99 (H3Y99sulf) in the cytoplasm, catalyzed by the cytosolic sulfotransferase SULT1B1. The modified H3 binds H4, enters the nucleus, and integrates into active chromatin, where H3Y99sulf is proposed to recruit PRMT1 to induce H4R3me2a and activate transcription. H3Y99sulf may contribute to several diseases. In HCC, hypoxia activates the Snail pathway, upregulating PAPSS2 to increase PAPS supply, thereby driving H3Y99sulf and promoting cancer progression. In gastric cancer, CAF-derived SEMA7A binds ITGB1 on cancer cells, activating the ERK1/2-CEBPB axis, which upregulates GAL3ST1. GAL3ST1 then is proposed to sulfate H3Y99. With AP2B1, H3Y99sulf enters the nucleus, recruits KAT2A to establish H3K56ac at the β-catenin promoter, and induces EMT, as reported. Beyond cancer, H3Y99sulf has been suggested to serve as an endogenous protective response in ischemic stroke. The SLC26A1-PAPSS1-SULT1B1 signaling cascade is activated during ischemic injury, increasing H3Y99sulf, which enhances glycolysis and metabolic gene expression to sustain neuronal survival. Thus, H3Y99sulf represents a candidate cytoplasmic histone modification with proposed key roles in transcription, cancer, and stress adaptation.

Although only a few sulfated proteins have been identified so far, PTS is a widespread post-translational modification that could theoretically modify not only membrane proteins but also other types. For example, a recent study reported the candidate occurrence of histone H3 tyrosine sulfation in the cytoplasm, rather than in the Golgi apparatus as traditionally believed [67]. As the fundamental structural components of chromatin, histones package genomic DNA and frequently undergo various PTMs. These histone modifications serve as pivotal epigenetic codes that govern chromatin remodeling and precisely regulate multiple core cellular events, including gene transcription, DNA replication, genome stability maintenance and DNA damage repair [189,190,191]. Histone sulfation represents a putative novel type of histone modification: the 99th tyrosine residue of histone H3 can be sulfated (H3Y99sulf). In the cytoplasm, SULT1B1, a member of the cytosolic sulfotransferase family (SULTs), directly uses PAPS as a donor to perform PTS modification on nascent histone H3 monomers. With chaperone assistance, H3Y99sulf forms a heterodimer with H4, enters the nucleus, and is incorporated into chromatin nucleosomes in transcriptionally active regions. There, H3Y99sulf is exposed on the nucleosomal surface, recruits PRMT1, and induces H4R3me2a modification in surrounding regions, thereby activating gene transcription (Figure 4) [67]. Furthermore, the research team discovered that H3Y99sulf also plays a potential critical role in the progression of hepatocellular carcinoma (HCC). Mechanistically, in the hypoxic tumor microenvironment—a hallmark of solid tumors—the Snail signaling cascade is persistently activated in HCC cells. This activation upregulates the transcriptional expression of PAPSS2, a key synthetase responsible for intracellular PAPS biosynthesis. Elevated PAPSS2 expression augments cellular PAPS abundance, which provides sufficient sulfate donors to sustain robust H3Y99sulf modification. Functionally, the accumulated H3Y99sulf initiates and sustains the H3Y99sulf-H4R3me2a-TDRD3 signaling axis, which activates the transcription of target genes associated with tumor metabolic reprogramming and hypoxic adaptation. This oncogenic signaling cascade was proposed to drive malignant proliferation and progression of HCC (Figure 4) [192]. The pro-tumorigenic activity of H3Y99sulf is not limited to HCC, as it also mediates malignant progression in gastric cancer via a distinct microenvironment-dependent regulatory mechanism. Cancer-associated fibroblasts (CAFs) within the tumor stroma actively communicate with cancer cells, creating a unique microenvironment that fosters tumor progression [193,194]. In gastric cancer, CAFs secrete SEMA7A ligands, which bind to the transmembrane receptor ITGB1 on gastric cancer cell surfaces. This ligand–receptor interaction triggers the phosphorylation and activation of the ERK1/2-CEBPB signaling pathway, thereby transcriptionally upregulating the expression of galactose-3-O-sulfotransferase 1 (GAL3ST1), a key sulfotransferase. Cytoplasmic GAL3ST1 further catalyzes the site-specific sulfation of nascent histone H3 at the Y99 residue to generate functional H3Y99sulf. Subsequent nuclear transport of H3Y99sulf is mediated by the adaptor protein AP2B1. Within the nucleus, H3Y99sulf recruits the histone acetyltransferase KAT2A to the promoter region of β-catenin, promoting H3K56ac and epigenetic activation of β-catenin transcription. The hyperactivated β-catenin signaling further initiates epithelial–mesenchymal transition (EMT), a core process driving tumor invasion and metastasis in gastric cancer (Figure 4) [68]. Beyond its known role in tumors, upregulation of H3Y99sulf has now been identified as a candidate response mechanism of the brain to the onset of ischemic stroke. Under ischemic conditions, the SLC26A1-PAPSS1-SULT1B1 signaling axis is activated, leading to increased H3Y99sulf. This upregulated H3Y99sulf in turn promotes the transcription of metabolic genes and enhances glycolysis, thereby sustaining cell survival under ischemic stress (Figure 4) [195].

Nevertheless, H3Y99sulf has also sparked ongoing debate. Youssef et al. challenged prior claims of histone tyrosine sulfation based on their negative MS and in vitro enzyme assay results [196], whereas Bai et al. attributed the observed discrepancies to variations in sample preparation and assay conditions [90]. Collectively, mass spectrometry faces multiple inherent technical challenges for PTS detection. Given these limitations, MS alone cannot provide definitive proof for PTS. Therefore, biochemical validation, alongside MS evidence, is essential to confirm PTS within the mammalian proteome. We anticipate that, with technological advances, emerging approaches such as an engineered protein nanopore developed by Niu and colleagues may help resolve these technical challenges and facilitate PTS detection [90,197].

In summary, the discovery of H3Y99sulf expands the landscape of canonical histone modifications and establishes histone sulfation as a novel, functionally indispensable epigenetic regulatory mechanism. The context-dependent functions of H3Y99sulf in tumor malignancy and cerebral ischemic stress response demonstrate its pleiotropic biological roles in both pathological and adaptive physiological processes. Collectively, these emerging findings not only deepen our mechanistic understanding of chromatin dynamics and epigenetic transcriptional regulation but also provide a novel conceptual framework for subsequent investigations on protein sulfation modifications and their disease-associated functions.

#### 4.2.2. p53

**Figure 5:**
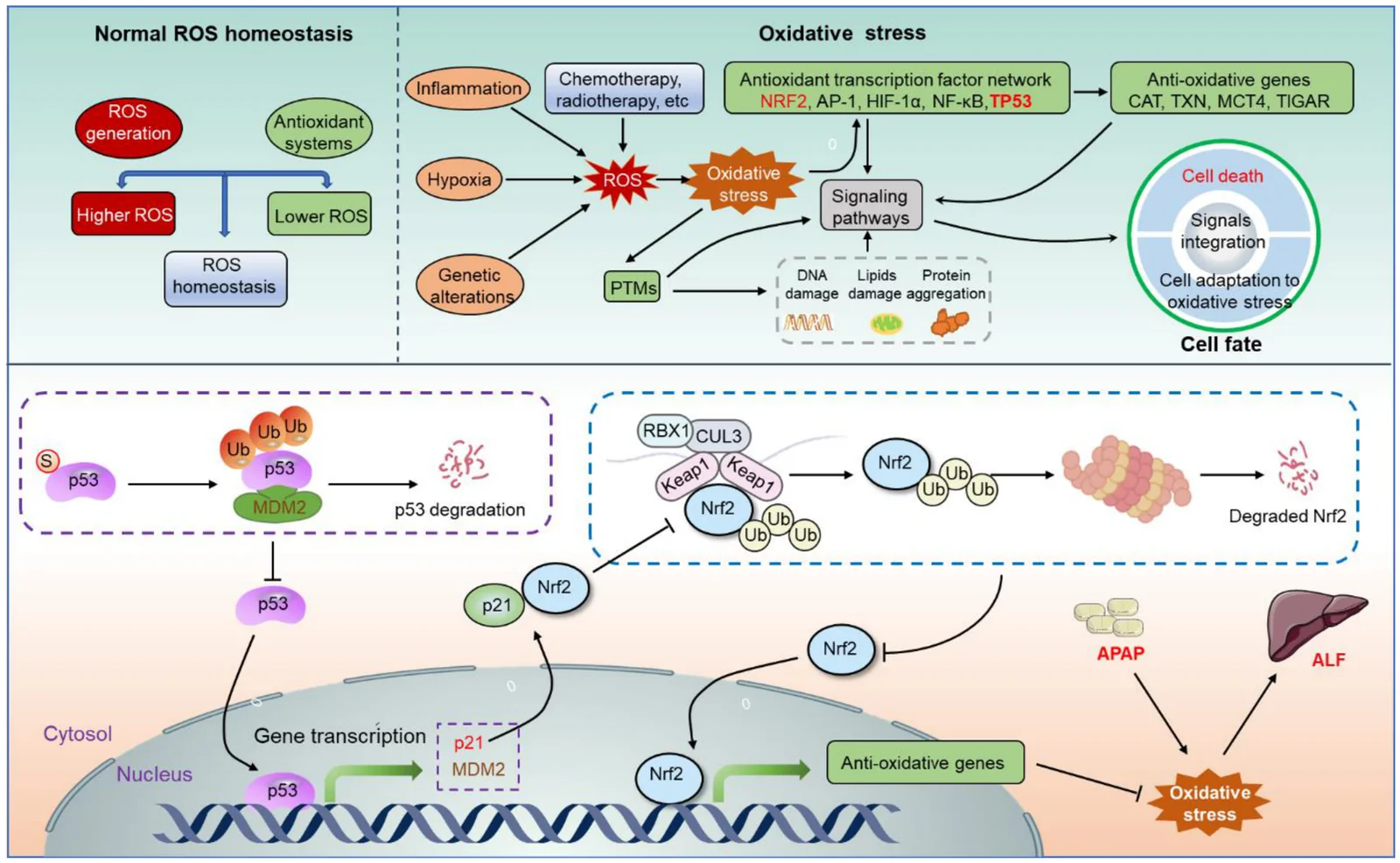
p53 tyrosine sulfation and its functional role in APAP-induced ALF. Under normal conditions, ROS homeostasis is maintained through continuous generation and elimination, allowing cells to avoid oxidative damage while supporting normal function. Beyond its cytotoxic effects on biological macromolecules, ROS also function as crucial intracellular signaling messengers, enabling cells to decipher and respond to redox signals to shape diverse cellular phenotypes. In APAP-induced ALF, oxidative stress drives pathogenesis. Notably, p53 undergoes tyrosine sulfation, which exacerbates liver injury. p53 stability is normally regulated by MDM2-mediated ubiquitination and degradation. Inhibiting p53 sulfation disrupts this interaction, leading to p53 accumulation. The accumulated p53 further activates the p53-p21-Nrf2 antioxidant signaling axis, which serves as a compensatory defensive mechanism to mitigate APAP-elicited oxidative stress and hepatic damage. Collectively, p53 sulfation exacerbates oxidative injury during ALF progression, rendering it a promising therapeutic target for the clinical intervention of APAP-induced liver damage.

Beyond histone substrate, recent research has further extended the regulatory spectrum of PTS modification by identifying p53 as a novel PTS substrate that mediates acetaminophen (APAP)-induced acute liver failure (ALF) [99]. Encoded by the *TP53* gene, p53 is a canonical transcription factor and well-characterized tumor suppressor, widely recognized as the “guardian of the genome” due to its central governance over cellular homeostasis. The transcriptional activity of p53 is indispensable for modulating multiple fundamental biological processes, including cell cycle checkpoint control, programmed cell death, and DNA damage repair [198,199]. Under physiological conditions, p53 maintains a low basal expression level, whereas diverse cellular stress stimuli can rapidly trigger its upregulation and functional activation [200]. As a short-lived protein, its stability, subcellular localization, and transcriptional activity are frequently regulated by various PTMs. These modifications are crucial for p53 activation and are closely linked to specific p53 regulatory proteins such as MDM2 and MDM4/MDMX [201]. Mechanistically, MDM2 functions as a canonical E3 ubiquitin ligase that directly catalyzes the ubiquitination of p53, thereby targeting p53 for proteasome-dependent degradation to constrain its cellular abundance under basal conditions [202]. Notably, MDM2 itself is a direct transcriptional target of p53, forming a classic negative feedback loop that allows for tight regulation of p53 signaling and maintains cellular p53 homeostasis in unstressed states [203,204]. In response to mild or transient acute stress, activated p53 serves as a core protective regulator by scavenging excessive reactive oxygen species (ROS), mitigating oxidative damage, and facilitating cellular repair and survival. Conversely, under conditions of severe or persistent stress, sustained p53 activation alternatively drives cellular senescence or apoptotic programs to eliminate irreversibly damaged cells, preventing the accumulation of defective cells and maintaining tissue integrity [205,206]. In the context of APAP-ALF, p53 sulfation exerts a distinct pathogenic role in liver injury. Functional experimental evidence has validated that p53 sulfation acts as a pro-injury modification to aggravate hepatic damage during APAP intoxication. Genetic or pharmacological inhibition of p53 sulfation in mouse models effectively disrupts the physical interaction between p53 and MDM2, thereby blocking MDM2-mediated ubiquitination and subsequent proteasomal degradation of p53. The resultant stabilization and intracellular accumulation of functional p53 further activate the downstream p53-p21-Nrf2 antioxidant signaling axis, which markedly enhances hepatic resistance to APAP-induced oxidative toxicity and alleviates liver injury. In aggregate, this pioneering study uncovers a novel PTS-dependent regulatory mechanism governing p53-mediated oxidative stress responses in the liver and highlights the therapeutic potential of targeting p53 sulfation as an innovative intervention strategy for the clinical treatment of APAP-induced ALF (Figure 5) [99].

### 4.3. Cytoplasmic Proteins

#### SAV1

**Figure 6:**
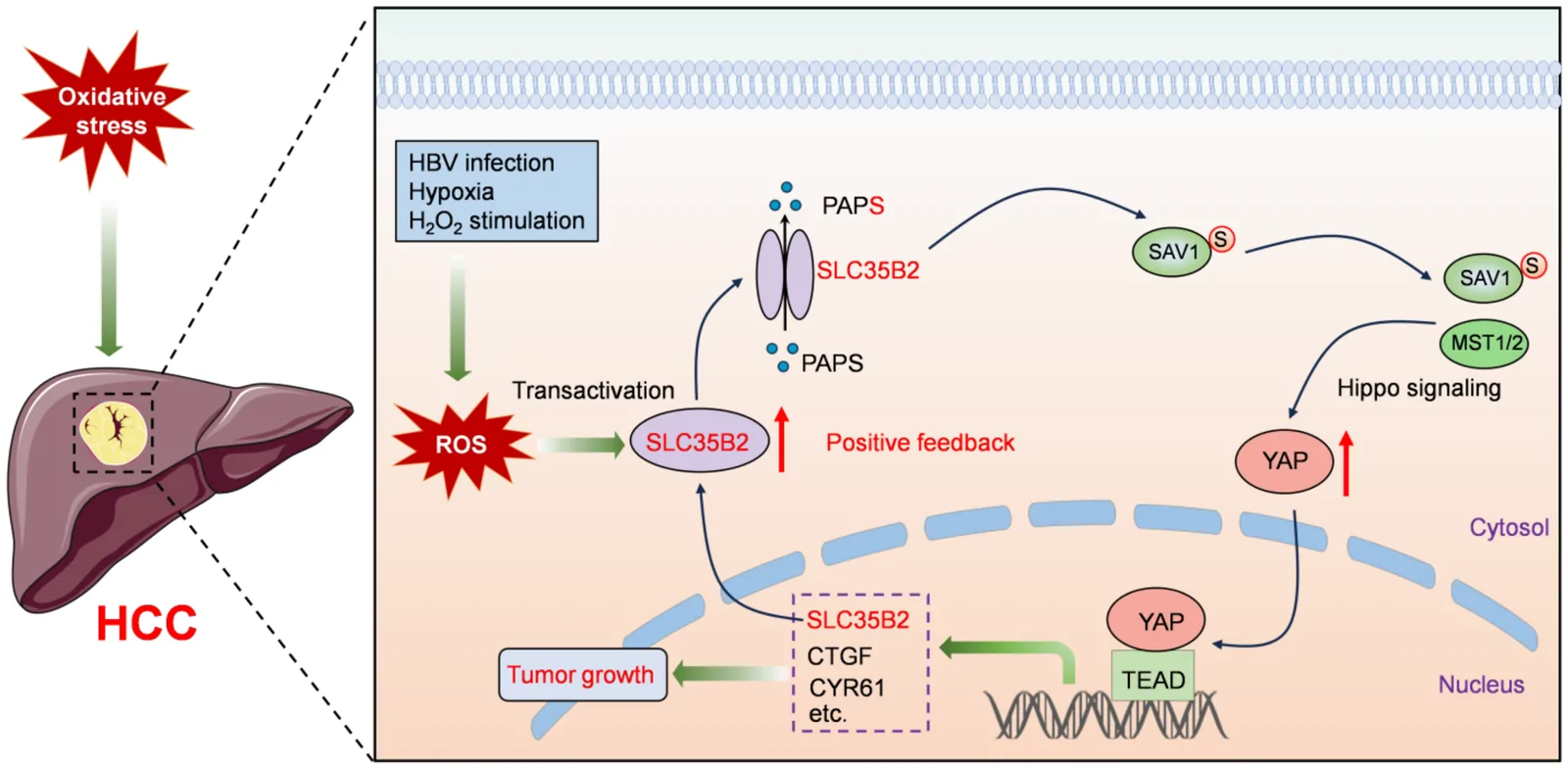
SLC35B2/SAV1 sulfation axis in HCC. In HCC, oxidative stress upregulates SLC35B2 expression, which delivers PAPS to facilitate SAV1 tyrosine sulfation. An unresolved question remains how cytosolic SAV1 enters the Golgi lumen to undergo TPST-mediated sulfation. Sulfated SAV1 loses the capacity to form functional homodimers, impairing MST1-SAV1 complex assembly and subsequent MST1 kinase activation. Such dysfunction ultimately inactivates the Hippo tumor-suppressive pathway and facilitates HCC malignant progression. Moreover, nuclear accumulation of activated YAP further transcriptionally upregulates SLC35B2, establishing a positive feedback loop that accelerates HCC deterioration. Collectively, SAV1 sulfation functions as a proposed critical pro-tumorigenic modification in HCC. The SLC35B2/SAV1 regulatory axis provides valuable candidate biomarkers and actionable therapeutic targets for HCC clinical treatment.

The Hippo signaling pathway is a highly conserved and pivotal regulatory cascade that has attracted extensive research attention in oncology, owing to its indispensable function in modulating tumor initiation and progression [207,208]. This pathway dominates a series of fundamental cellular biological processes, including proliferation, differentiation, and apoptosis, thereby contributing to the precise regulation of organ size and tissue homeostasis in physiological conditions [209]. The canonical Hippo pathway consists of a well-orchestrated kinase cascade. Upon activation, the MST1/2-SAV1 complex directly phosphorylates and activates the LATS1/2-MOB1A/1B complex, which in turn phosphorylates the downstream transcriptional effectors YAP and TAZ. This phosphorylation event prevents YAP/TAZ from translocating into the nucleus and promotes their ubiquitination-dependent degradation, thereby suppressing their oncogenic activity. In this sophisticated kinase cascade, SAV1 and MOB1A/1B act as indispensable scaffolding and adaptor proteins that enhance the kinase activities of MST1/2 and LATS1/2, respectively [210]. Given the definitive tumor-suppressive property of the Hippo pathway in most solid tumors, dysregulation of its core components is tightly implicated in malignant transformation and tumor progression. Among these components, SAV1 has recently been identified as a novel substrate of tyrosine sulfation, harboring multiple putative sulfation sites, and plays a significant role in the progression of hepatocellular carcinoma (HCC). In HCC, SAV1 sulfation is markedly upregulated and positively correlates with the expression of SLC35B2, a transporter responsible for supplying the sulfate donor PAPS. Mechanistically, oxidative stress, a hallmark pathological feature of HCC microenvironment, induces the transcriptional upregulation of SLC35B2, which subsequently promotes the sulfation of SAV1. Structurally, normal SAV1 requires homodimerization to form a stable protein conformation, which is a structural prerequisite for the assembly of the MST1-SAV1 complex and subsequent activation of MST1 kinase activity. However, tyrosine sulfation disrupts the functional homodimers of SAV1, leading to impaired formation of the MST1-SAV1 complex. The disassembly and functional inactivation of the core upstream kinase complex ultimately block the transduction of Hippo tumor-suppressive signaling, resulting in the aberrant activation of downstream oncogenic YAP/TAZ signaling and driving the malignant progression of HCC. Notably, a positive feedback regulatory loop is formed during this pathological process. The aberrantly activated YAP, the major downstream effector of the inactivated Hippo pathway, can further bind to the promoter region of SLC35B2 and transcriptionally upregulate SLC35B2 expression. This regulatory loop continuously amplifies SAV1 sulfation, persistently suppresses Hippo tumor-suppressive signaling, and further accelerates the malignant evolution of HCC (Figure 6) [211]. However, the mechanism by which cytosolic SAV1 gains access to the Golgi lumen to undergo TPST-mediated PTS remains unresolved. Collectively, these findings reveal a pro-tumorigenic role of tyrosine sulfation modification in HCC, deepen our understanding of the malignant evolution of this cancer type, and offer potential biomarkers or therapeutic targets for clinical intervention.

### 4.4. Other Proteins

Despite the fact that only a small number of sulfated proteins has been successfully identified and functionally validated via experimental approaches to date, PTS is increasingly recognized as a highly conserved, prevalent, and functionally versatile PTM across eukaryotic organisms. In theory, this modification is not restricted to specific protein subsets and can occur on a broad spectrum of protein substrates, implying its far-reaching biological potential. Indeed, studies have shown that, in addition to the previously mentioned protein categories, certain secreted proteins, such as progastrin, thyroglobulin, and pancreatic trypsinogen, also undergo tyrosine sulfation. These secreted proteins possess evolutionarily conserved tyrosine sulfation motifs, and PTS serves as a crucial molecular switch that modulates their post-translational proteolytic processing, structural maturation, and ultimate biological functionality [212,213,214]. As a well-characterized example, PTS modification facilitates proteolytic processing of progastrin, thereby governing its biological potency in regulating gastrointestinal physiological functions [201]. This finding indicates that tyrosine sulfation not only regulates protein stability and function but also plays a critical role in the maturation of hormones and enzymes. Importantly, PTS is not restricted to the animal kingdom. Accumulating evidence suggests that PTS modification also extensively exists in plant and microbial proteins, executing diverse and essential physiological functions in distinct biological contexts [26,215,216,217,218,219,220,221]. In plant systems, for instance, multiple secreted peptide hormones, such as PSKs, PSYs, CIFs, and RGFs, rely strictly on PTS to trigger downstream signaling cascades. Such PTS-dependent activation of peptide hormones is fundamental to modulating plant growth, developmental patterning, and biotic or abiotic stress tolerance responses throughout the plant life cycle [209]. These cross-species discoveries collectively demonstrate that PTS constitutes an ancient and evolutionarily conserved PTM mechanism, whose biological functions and regulatory breadth are substantially broader than previously recognized.

Although the functional characterization and mechanistic exploration of PTS remain in continuous advancement, its ubiquitous distribution across animals, plants, and microorganisms strongly suggests that the actual prevalence, substrate diversity, and functional complexity of PTS are considerably underestimated by current research. Taken together, the accumulating evidence indicates that PTS likely carries out regulatory functions of universal biological significance, broadly participating in key physiological and pathological processes, including secretory pathways, signal transduction, developmental regulation, and host–pathogen interactions. Future research should focus on the systematic identification of additional sulfated protein substrates and the elucidation of their molecular mechanisms, combining high-resolution mass spectrometry with bioinformatic predictions, an important direction for advancing this field.

## 5. Conclusions

PTS represents a prevalent and functionally vital PTM in eukaryotic organisms, which is catalyzed by TPSTs. These enzymes specifically mediate the covalent transfer of activated sulfate groups from the universal sulfate donor PAPS onto the tyrosine residues of substrate proteins. Since the initial discovery of PTS in 1954, numerous sulfated proteins have been identified. Early studies predominantly confined PTS modification to secreted and transmembrane proteins, which were considered the primary substrates of this PTM. However, emerging evidence has substantially broadened the substrate spectrum of PTS, confirming the existence of functional PTS modifications in cytoplasmic and nuclear proteins, thereby expanding the subcellular distribution of this regulatory mechanism. Functionally, PTS participates in a wide array of fundamental physiological processes and pathological events across human systems, including blood coagulation, leukocyte recruitment and adhesion, complement cascade activation, hormone maturation and signaling regulation, viral invasion and infection, as well as tumor initiation and malignant progression. At the molecular level, the versatile biological functions of PTS are primarily attributed to its unique capacity to reshape protein surface conformation and modulate dynamic protein–protein interactions. Beyond intermolecular binding regulation, PTS modification also exerts profound regulatory effects on the intrinsic bioactivity, proteolytic processing maturation, and clinical therapeutic responsiveness of target proteins, serving as a multi-dimensional molecular regulator of protein function. Despite growing understanding of PTS in recent years, many mysteries in this field remain to be unraveled. First, it is still unclear what proportion of the human proteome can undergo PTS modification. Second, most of its biological functions remain unknown. Compounding these challenges, accurate detection of PTS is difficult due to its instability and the fact that the associated molecular weight increase closely resembles that of phosphorylation. To date, only a few PTS-modified proteins have been functionally characterized, while the upstream regulatory triggers and downstream functional cascades of most PTS substrates remain elusive. Furthermore, it remains unknown how PTS-related enzymes and transporters respond to environmental changes, whether PTS exhibits cell- or tissue specificity, and how TPST-1 and TPST-2 differ in substrate specificity, expression, and function. Resolving these key issues urgently requires the development of highly sensitive, modification-specific detection technologies, coupled with optimized bioinformatic algorithms for precise sulfation site prediction, which will facilitate the large-scale discovery and functional verification of novel PTS substrates.

Despite the existing technical and mechanistic challenges, PTS has emerged as a promising target for translational medicine and therapeutic development, with increasing evidence validating its clinical potential. Given that PTS directly modulates the binding affinity between cell surface receptors and their corresponding ligands, this modification serves as a critical determinant of signaling activation, making it an actionable target for novel therapeutic strategy development. A canonical and well-validated example is the tyrosine sulfation of CCR5. As an essential co-receptor mediating HIV-1 entry into host immune cells, CCR5 relies on tyrosine sulfation modification to achieve high-affinity binding with the viral envelope glycoprotein gp120. Based on this definitive molecular mechanism, researchers have engineered sulfated peptide analogs that mimic the sulfated functional domain of CCR5. These synthetic analogs competitively interact with gp120, thereby efficiently blocking the viral entry pathway and exerting robust anti-HIV activity in both in vitro cellular models and in vivo animal experiments, providing proof of concept for the development of PTS based antiviral drugs. More broadly, given that PTS plays a significant role in various diseases, PTS-targeted interventions could be extended to other infectious diseases and immune disorders. In addition to sulfated peptide mimicry strategies, multiple PTS-targeted therapeutic modalities have been proposed, including small-molecule inhibitors targeting key enzymes involved in the PTS modification cascade, specific drugs locking functional sulfation sites, and engineered antibodies or nanobodies that specifically recognize sulfated protein epitopes. Collectively, the feasibility of targeting PTS is being validated, marking a critical transition of PTS research from basic mechanistic exploration to clinical translational application.

Looking ahead, future research should focus on the following priorities. First, novel detection methods should be developed, or existing techniques optimized, to improve the sensitivity and accuracy of identifying target proteins and their sulfation sites. Second, in-depth translational research is urgently needed to develop diversified PTS-targeted therapeutic strategies, including sulfated peptide mimetics, specific small-molecule inhibitors, and modification-specific antibodies, based on the essential roles of PTS in physiological homeostasis and disease pathogenesis. In summary, continued in-depth investigation of the PTS biosynthetic pathway, elucidation of its dynamic changes and regulatory networks, and exploration of its associations with disease onset and progression are of great significance for understanding the fundamental principles of life and for developing novel precision therapeutics. We anticipate that future research in the PTS field will uncover further mysteries and exert a profound impact on therapeutic and preventive strategies for human diseases.

## Data Availability

No new data were created or analyzed in this study. Data sharing is not applicable to this article.

## Abbreviations

PTS: protein tyrosine sulfation; PTM: post-translational modification; PAPS: 3′-phosphoadenosine-5′-phosphosulfate; PAPSS: PAPS synthase; ATP: adenosine triphosphate; APS: adenosine-5′-phosphosulfate; TPSTs: tyrosylprotein sulfotransferases; TLC: thin-layer chromatography; SDS-PAGE: SDS-polyacrylamide gel electrophoresis; MS: mass spectrometry; GPCRs: G-protein-coupled transmembrane receptors; OSF: oral submucous fibrosis; FBs: fibroblasts; LMP1: Latent membrane protein 1; EBV: Epstein–Barr virus; NPC: nasopharyngeal carcinoma; PSGL-1:P-selectin glycoprotein ligand-1; EV71:enterovirus 71; GPCR: G protein-coupled receptor; *S. aureus*: *Staphylococcus aureus*; MRSA: methicillin-resistant strains; CHIPS; staphylococcal chemotaxis inhibitory protein; PFTs: pore-forming toxins; SCARB2: scavenger receptor class B member 2; ITGB4: integrin beta4; HCC: hepatocellular carcinoma; CAFs: cancer-associated fibroblasts; GAL3ST1: galactose-3-O-sulfotransferase 1; APAP: acetaminophen; ALF: acute liver failure; ROS: reactive oxygen species.
